# OPN‐Derived Peptides Generated by Proteasomes Can Promote Cell Migration via CD44 Activation

**DOI:** 10.1155/jimr/2726052

**Published:** 2026-02-25

**Authors:** Hindh Imad, Kathryn E. Strange, Chiara Dianzani, Wai Tuck Soh, Sophie Siddons, Shahram Kordasti, Casimiro Luca Gigliotti, Annalisa Chiocchetti, Henning Urlaub, Timothy James Nott, Umberto Dianzani, Juliane Liepe, Michele Mishto

**Affiliations:** ^1^ Research Group of Molecular Immunology, The Francis Crick Institute, London, NW1 1AT, UK, crick.ac.uk; ^2^ Peter Gorer Department of Immunobiology, King’s College London, London, SE1 1UL, UK, kcl.ac.uk; ^3^ Department of Drug Science and Technology, University of Turin, 10126, Torino, Italy, unito.it; ^4^ Research Group of Quantitative System Biology, Max-Planck-Institute for Multidisciplinary Sciences, 37077, Göttingen, Germany, uni-goettingen.de; ^5^ School of Cancer and Pharmaceutical Sciences, King’s College, London, UK, kcl.ac.uk; ^6^ Haematology Department, Guy’s Hospital, London, UK, nhs.uk; ^7^ Department of Clinical and Molecular Sciences, Università Politecnica delle Marche, Ancona, Italy, univpm.it; ^8^ Interdisciplinary Research Centre of Autoimmune Diseases (IRCAD), University of Piemonte Orientale, Amedeo Avogadro, 28100, Novara, Italy, uniupo.it; ^9^ Department Health Sciences, Università Piemonte Orientale, Novara, Italy, uniupo.it; ^10^ Research Group of Bioanalytical Mass Spectrometry, Max-Planck-Institute for Multidisciplinary Sciences, 37077, Göttingen, Germany, uni-goettingen.de; ^11^ Department of Clinical Chemistry, University Medical Center Göttingen, 37075, Göttingen, Germany, umg.eu; ^12^ Department of Chemistry, King’s College London, London, SE1 1DB, UK, kcl.ac.uk; ^13^ Core Facility for Data Sciences and Biostatistics, Max-Planck-Institute for Multidisciplinary Sciences, 37077, Göttingen, Germany, uni-goettingen.de; ^14^ Centre for Inflammation Biology and Cancer Immunology, King’s College London, London, SE1 1UL, UK, kcl.ac.uk

**Keywords:** 20S proteasome, CD44, disordered proteins, inSPIRE, lymphocytes, migration, osteopontin, peptide splicing

## Abstract

Osteopontin (OPN) is a pleiotropic cytokine that is overexpressed in many autoimmune diseases and solid cancers. Here we show that the 20S proteasome can degrade, in vitro, both the full‐length OPN and the OPN‐C fragment generated in the extracellular space and produce both canonical and spliced peptides. Specific canonical OPN‐derived peptides, generated from hotspots within the OPN sequences, can regulate cell migration via CD44. By predicting peptide‐protein docking, we propose that this could occur by displacing the flexible C‐terminal region from the CD44 α1 helix, promoting a conformational change in CD44 that could lead to the activation of downstream signalling pathways. We also identify key amino acid residues within these OPN‐derived peptides that impact the activation of CD44 to initiate cell migration and predict their binding to the CD44 asparagine 164 residue—previously implicated in stabilising the CD44 structure to promote cell migration. Therefore, we propose that proteasomes can process various OPN isoforms, theoretically both inside and outside a cell, leading to the presence of OPN‐derived peptides in the extracellular space where they can play an immunological role. This process, which others showed to represent an antibacterial defence, could also be involved in the regulation of cell surface receptors.

## 1. Introduction

Osteopontin (OPN), a component of bone matrix and a soluble pleiotropic cytokine, plays a pivotal role in many diseases such as cancer, myocardial and kidney dysfunctions, rheumatoid arthritis and multiple sclerosis [1]. For example, in multiple sclerosis, OPN stimulates the expression of Th1 and Th17 cytokines, inhibits apoptosis of autoreactive T cells and regulates leukocyte adhesion, migration and trafficking into the central nervous system. These latter processes are mediated by binding to two classes of adhesion molecules, CD44 and various integrins, including α4β1 integrins [2, 3]. OPN has been shown to contribute to the regulation of physiological processes such as bone mineralisation and immune cell regulation, and its compromised expression could lead to immune system‐related diseases such as osteoarthritis, multiple sclerosis, systemic lupus erythematous and cancer [4–6]. It has been demonstrated that the interaction of OPN with CD44 can facilitate the activation of downstream intracellular signalling pathways that regulate cell proliferation, migration, stemness of cells and tumorigenesis [6]. OPN expression has been shown to upregulate NOX1 via the JAK2/STAT3 pathway, leading to elevated production of reactive oxygen species [6]. OPN activation of CD44 also regulates PI3K/Akt/mTOR and NF‐κB pathways in glioma cell lines and lipid injured hepatocytes, respectively [6–9]. Hyaluronan conventionally binds CD44, driving the rolling‐adhesion steps that promote lymphocyte migration, mediated by the CD44 conformational change [10].The rolling stage occurs by the alternation between the partially disordered state of CD44—promoting bond association at the leading edge—and its ordered state, promoting bond dissociation at the rear end between CD44 present on the endothelium cell membrane and the migrating cells [11, 12].

Secreted OPNs (sOPNs) have been shown to promote cell migration also via CD44 [13, 14]. Research has mainly focused on the function of secreted full length OPN (sOPN‐FL), despite an intracellular full length OPN form (iOPN‐FL) also being expressed. iOPN has been detected in the cytoplasm, localising at perinuclear and perimembranous surfaces [15]. On the contrary, sOPN‐FL contains an N‐terminal signal peptide sequence, which is cleaved upon secretion via secretory vesicles. In the extracellular space, sOPN‐FL molecules can be cleaved by matrix metalloproteinases and thrombin [1, 16]. Thrombin cleaves sOPN‐FL into N‐terminal (sOPN‐N), which contains an RGD motif essential for interacting with integrins, and C‐terminal (sOPN‐C) fragments (Figure S1), which has been hypothesised to interact with CD44 to exert different biological activities [13, 17]. Within the extracellular space, the 20S proteasome has been shown to process sOPN‐FL and its fragments, generating peptides that can promote cell migration [18, 19]. This is likely due to sOPNs being intrinsically disordered proteins (IDPs). IDPs are estimated to represent at least 30% of the human proteome and could be cleaved by 20S proteasomes via recognition of their intrinsically disordered regions (IDRs) [20–24]. Inside cells, the 20S proteasome is the core of the ubiquitin proteasome system (UPS), and when coupled to the 19S regulatory complex, forms the 26S that cleaves the majority of the cytoplasmic proteins in eukaryotic cells tagged by a poly‐ubiquitin chain. In the extracellular space, including the contents of extracellular vesicles (EVs), 20S proteasomes are theorised to be proteolytically active [18, 25–35] and the extracellular proteasome content is associated to many immunology‐related diseases [18, 19, 36–42].

Although the most known immunological function of the peptides produced by proteasomes is the presentation by human leukocyte antigen class I (HLA‐I) complexes and their recognition by CD8^+^ T cells, proteasomes can also activate transcription factors such as NF‐κB, Spt23p and Epe1 by partial processing of their precursors [43, 44]. Recently, proteasome‐mediated degradation of many human proteins has been hypothesised to be an important mechanism of defence against bacterial infection by the production of peptides that directly disrupt microbial cell membranes. These peptides produced by proteasomes inside cells can be released in the extracellular space, where they can mediate their anti‐bacterial activity [44]. Proteasomes can catalyse not only peptide hydrolysis, whereby canonical non‐spliced peptides are produced but also peptide splicing whereby two non‐contiguous fragments of a protein are ligated together [45]. Both processes occur in a regulated fashion driven by peptide sequence preferences and other factors [24, 46–51].

Here, we investigated the molecular mechanism promoting the migration of peripheral blood lymphocytes (PBL) and other human cells via CD44, mediated by OPN‐derived peptides that can be produced by 20S proteasome. We propose that this could be a more general regulatory pathway of immunology‐related processes carried out by human cells via the regulation/activation of cell membrane receptors by 20S proteasome‐derived peptides.

## 2. Results

### 2.1. 20S Proteasomes Can Produce Both Canonical and Spliced Peptides During the Processing of OPNs

We previously identified peptides produced by 20S proteasomes during the processing of sOPN‐FL and sOPN‐C (Table S1) in the extracellular space; some of them stimulated the migration of PBLs, monocytes and human umbilical vein endothelial cells (HUVECs) [18]. For a more detailed and quantitative analysis of the peptide products generated by 20S proteasomes whilst processing sOPNs we applied our recently published in vitro–in silico pipeline to new sOPN‐FL in vitro digestion, which allows the identification of both canonical and spliced peptides [24]. Human 20S standard proteasomes were able to process sOPN‐FL, the peptide products were measured by mass spectrometry (MS) and the data was analysed by inSPIRE 1.5—aSPIRE software (Figure 1A). This software optimises the identification of both canonical and spliced peptides, limits the false sequence assignments (also using a proteomics‐informed contamination database), quantifies the peptide products and their abundance, generates peptide coverage maps and provides several quality check parameters to evaluate the outcome [24] (Figure 1A). From the proteasome‐mediated degradation of the recombinant sOPN‐FL, we identified a total of 1054 unique peptides by applying inSPIRE 1.5, wherein 952 were canonical peptides and 102 spliced peptides (Figure 1B), identified with high quality parameters of the peptide spectrum matches (PSMs; Figure S2A). Of all PSMs detected, the abundance of canonical peptides was considerably higher than that of spliced peptides (1.6% of the total; Figure 1C), which was in line with previous accounts for peptide identification [24]. The production of canonical and spliced peptides was concentrated in sOPN‐FL hotspots (Figure 1D), which were consistent over time (Figure S2B). Among the peptide products of sOPN‐FL, and within a hotspot, we identified the OPN_217–230_ peptide, which was already proved to be produced in vitro by 20S proteasomes and able to stimulate cell chemotaxis in HUVECs, PBL and monocytes [18]. The peptide was detected as such, as well as N‐terminally and C‐terminally extended peptides (Figure S3A, Table S2). We considered also peptide trimming in our analysis because antigenic peptides can be generated as precursors by proteasomes and then trimmed at both N‐ and C‐termini by aminopeptidases both in the cytosol and in the endoplasmi reticulum [23]. To determine the reliability of OPN_217–230_ production by 20S proteasomes, OPN‐C substrate (sOPN‐C_a_) was newly synthesised and digested, and the previously published sOPN‐C (sOPN‐C_b_) and sOPN‐N digestions were re‐analysed (Table S1), using the inspire 1.5—aSPIRE pipeline [18]. The OPN_217–230_ peptide was detected as such also in both sOPN‐C_a_ and sOPN‐C_b_ substrates (Figure S3B, Table S2A). Irrespective of synthesis variations and differences in mass spectrometer measurements (see Section 4 for details) the OPN_217–230_ peptide was consistently detected, thereby confirming the reproducibility of the peptide production and of its identification by MS. As expected, it was not identified among the peptide products of the proteasome‐catalysed in vitro digestion of the sOPN‐N substrate, which does not contain this peptide sequence (Table S1).

Figure 1Prevalence of canonical and spliced peptides produced by 20S proteasomes whilst processing OPN proteins in vitro. (A) Graphical representation of the peptide identification analysis performed in this study. 20S proteasome was used to digest OPNs in vitro and OPN‐derived peptides were measured by mass spectrometry (MS). Results were analysed using the inSPIRE 1.5—aSPIRE pipeline for the identification of canonical and spliced peptides. (B,C) Relative abundance (B) and number of unique (C) canonical and spliced peptides. Values were generated by applying inSPIRE 1.5 software to sOPN‐FL digestion and are merged and generated using aSPIRE software. For each substrate digestion there were two biological replicates. (D) Protein coverage profiles of canonical and spliced peptide products of in vitro digestions of sOPN‐FL with 20S proteasomes, with the locations of OPN_217–230_ and OPN_292–306_ annotated. Plot represents merged biological replicates (*n* = 2) (E) The coverage of canonical peptides from sOPN‐FL is overlaid with the IUPRED3 score which predicts the presence of disordered residues. The dashed line represents the score = 0.4, which is used as a threshold for an IDR. OPN enumeration refers to the sOPN‐FL sequence in Uniprot database (P10451).

**Figure:**
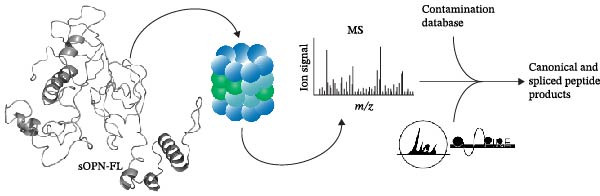
(A)

**Figure:**
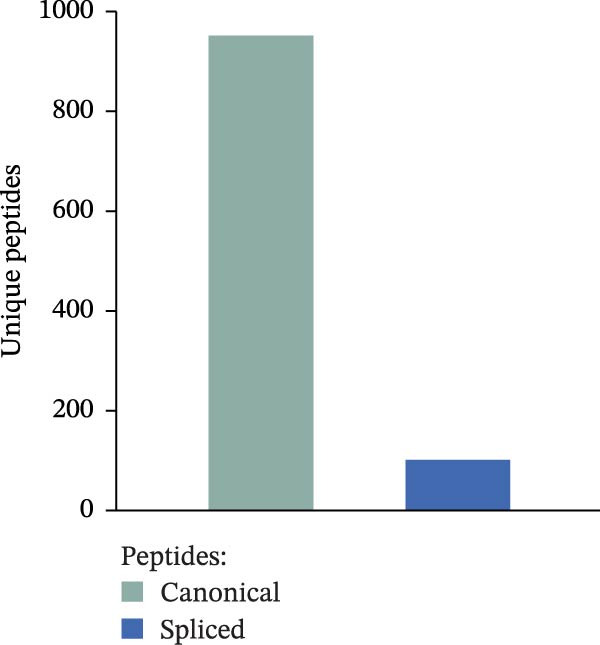
(B)

**Figure:**
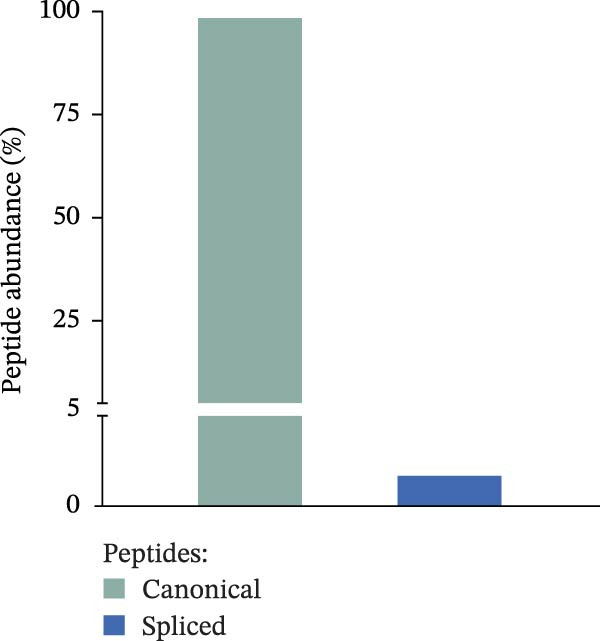
(C)

**Figure:**
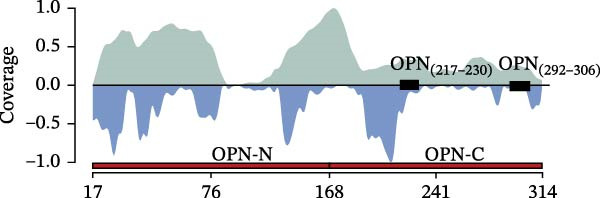
(D)

**Figure:**
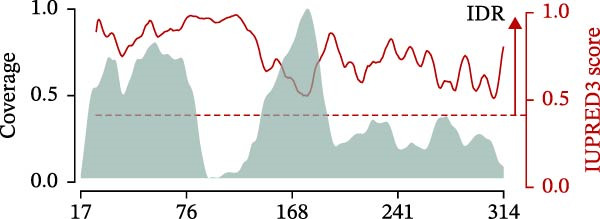
(E)

Another peptide hypothesised to stimulate cell chemotaxis was the OPN_292–306_ peptide [18], which in our OPN‐C digestion experiments was identified as such in the sOPN‐FL in vitro digestions with 20S proteasomes and as terminally extended peptides in the in vitro digestions of sOPN‐FL, sOPN‐C_a_ and sOPN‐C_b_ substrates (Table S2B).

Both OPN_217–230_ and OPN_292–306_ peptides were located in proteolysis hotspot regions of sOPN‐FL (Figure 1D), which were not obviously associated with the sOPN‐FL IDRs (Figure 1E).

It is still unknown if endoproteases can process OPNs either inside a cell, in EVs or in the extracellular and extra‐EV space. As a proof of concept, we investigated whether two main endoproteases, trypsin and chymotrypsin, could generate the OPN_217–230_ and OPN_292–306_ peptides. Both endoproteases could efficiently cleave all sOPNs although did not produce the 2 investigated peptides (Table S3).

### 2.2. The Proteasome‐Generated OPN_217–230_ and OPN_292–306_ Peptides Can Stimulate Cell Migration via CD44 Activation Without Acting on Cell Adhesion

In previous studies, we demonstrated that a handful of peptides produced by the 20S proteasome whilst processing sOPNs were capable of stimulating the migration of HUVECs, human PBLs and monocytes in a similar fashion to the intact sOPN‐FL and sOPN‐C proteins [18, 19]. CD44 is known to interact with sOPN‐C and promote cell migration [2, 52], hence CD44 could be involved in the cell chemotaxis triggered by proteasome‐generated OPN‐derived peptides. To test this hypothesis, we first assessed HUVEC migration—using Boyden’s migration chamber—in presence of a peptide pool derived from 20S proteasome‐catalysed degradation of sOPN‐C_b_ and an antibody blocking CD44 (Figure 2A). The proteasome‐generated sOPN‐C‐derived peptides significantly stimulated HUVEC migration and, by blocking CD44, the stimulation was significantly inhibited (Figure 2B). The CD44 inhibition by anti‐CD44 antibodies did not affect normal cell migration by HUVECs in our in cellula system, which, on the contrary, was inhibited by an anti‐integrin β1 antibody (Figure S4A). This occurred even in the presence of VEGF (Figure S4B), a known migratory stimulant, thereby confirming that the stimulated HUVEC migration triggered by the sOPN‐C‐derived peptides was specifically acting via CD44 activation (Figure 2B).

Figure 2The OPN_217–230_ and OPN_292–306_ peptides promote cell migration via CD44 receptor activation. (A) Boyden (BD Sciences) Chamber migration assay. (B, C) HUVEC migration stimulated by proteasome‐derived sOPN‐*C*_b_ peptide pool, VEGF (B) or OPN_217–230_ or OPN_292–306_ (C) ± antagonist antibodies blocking CD44. Values are percentage of treated vs. untreated cells that migrated after 20 h and are the mean and the SD of independent experiments (B: *n* = 7–39, C: *n* = 7–13). Cell migration was measured in the Boyden chamber migration assay. (D) 3D structure of the ordered (blue) and partially disordered (red) extracellular portion of CD44 (PDB: 1POZ). C terminal of CD44 connects to the CD44 transmembrane domain. (E) Relative PBL adhesion, in plates coated with hyalyronan, in presence of either OPN_217–230_, OPN_292–306,_ their inverted sequences or 1 ng/mL PMA. Values are percentage of adherent cells after 3 h treatment and are the mean and the SD of independent experiments (E: *n* = 9–21). Dots represent the single experiment values. Normality was determined in (B, C, E) using the Shaprio‐Wilk test. In (B, C, E), statistically significant difference between groups was tested by applying either Kruskal–Wallis test (B, C) or Tukey’s multiple comparisons test (E). Only significant *p*‐values are shown.

**Figure:**
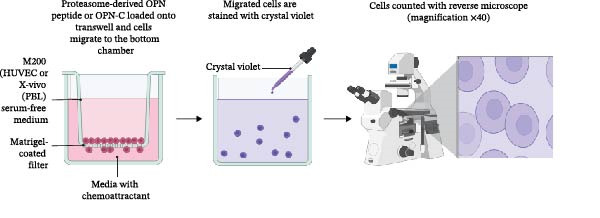
(A)

**Figure:**
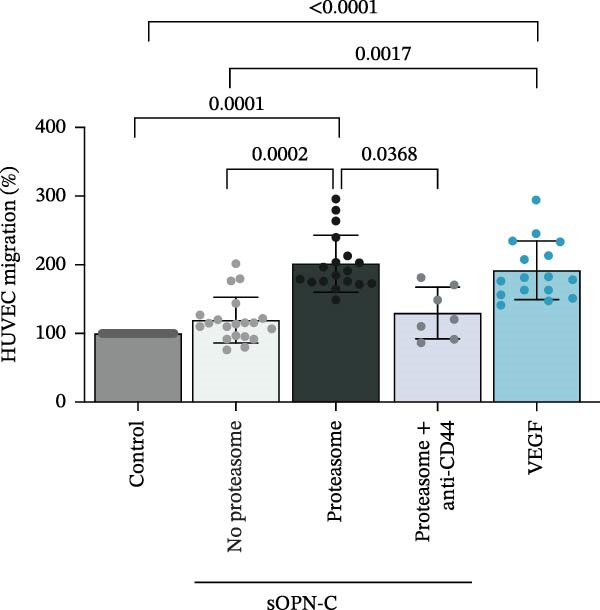
(B)

**Figure:**
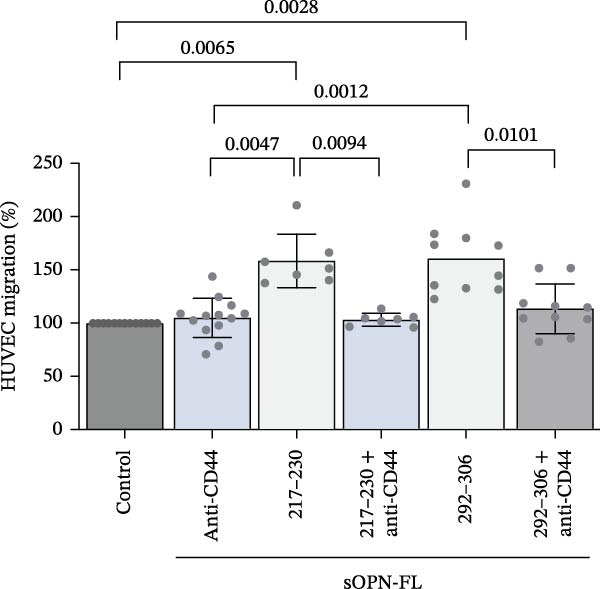
(C)

**Figure:**
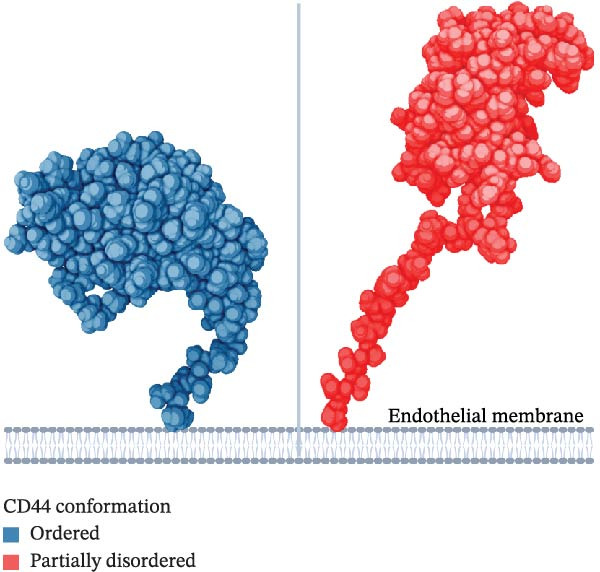
(D)

**Figure:**
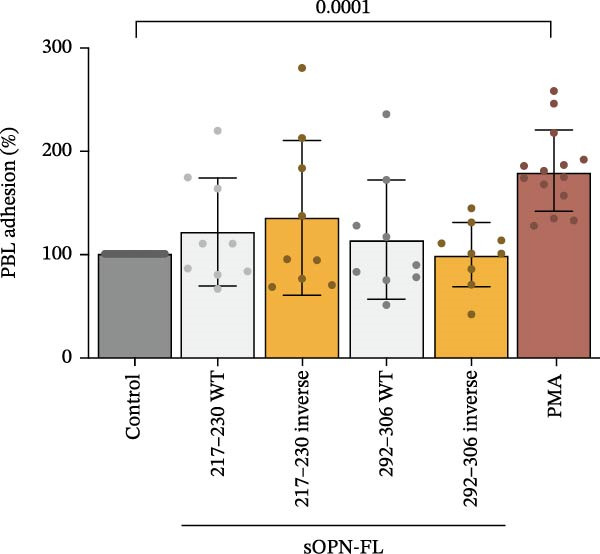
(E)

We then focused on the two selected OPN_217–230_ and OPN_292–306_ peptides, which we proved could be produced by 20S proteasome both from sOPN‐FL and sOPN‐C. To simplify, we will refer to them as derived from sOPN‐FL. We initially confirmed that these peptides significantly stimulated HUVEC migration and demonstrated that, by blocking CD44, the stimulation could be significantly inhibited (Figure 2C), thereby suggesting that the effect of these two peptides—and not only the whole peptide products of sOPN‐C digestion (Figure 2B)—on HUVEC migration was mediated by CD44.

The CD44‐mediated cell migration can be catalysed by a transition between the ordered and partially disordered conformation of CD44 (Figure 2D). This change in conformation may promote not only the activation of intracellular signalling pathways but also contribute to the structural requirements for hyaluronan‐mediated cell migration. Therefore, we investigated whether the OPN_217–230_ and OPN_292–230_ peptides could specifically promote the adhesion step of PBLs in presence of hyaluronan, which has been shown to be associated specifically with the partially disordered state of CD44 (Figure S5). Both peptides had no effect on PBL adhesion, neither as such nor as inverse sequence, which we used as control of the possible specificity of the activation process (Figure 2E).

### 2.3. Prediction of OPN_217–230_ and OPN_292–306_ Peptides Interaction to the Extracellular Portion of CD44

For an initial investigation of the possible molecular interaction between the OPN_217–230_ and OPN_292–306_ peptides and CD44, and how that might promote the cell migration, we carried out docking prediction of OPN_217–230_ and OPN_292–306_ peptides to the extracellular portion of CD44 (PDB: 1POZ) using CABS‐dock [53]. The ordered conformation of CD44 was primarily used to generate peptide structures, as it allowed for the flexibility of the tail portion of CD44 in similar fashion to that described by Takeda et al. [54], which showed the dynamics of the CD44 C‐terminal tail with and without hyaluronan.

Docking predictions would highlight potential binding regions, and so we hypothesise that these interactions could disrupt electrostatic interactions that stabilise the ordered CD44 conformation. The docking prediction using the ordered (PDB: 1POZ) conformation assigned the OPN‐derived peptides to near the C‐terminal flexible tail of CD44 (CD44_151–178_), indicating the peptides might impact the conformation of the flexible tail driving the activation of CD44 (Figure 3A–C).

Figure 3The predicted binding of OPN_217–230_ and OPN_292–306_ peptides to the ordered conformation of CD44. (A, B) Prediction of OPN_217–230_ (A) or OPN_292–306_ (B) docking to the ordered conformation of extracellular portion of CD44 (PDB: 1POZ). Both OPN peptides are predicted to interact with S_43_, N_164_, D_167_ and V_178_ residues of CD44. (A) OPN_217–230_ is predicted to bind the CD44 residues with its R_220_, S_228_ and E_226_ residues, respectively. (B) OPN_292–306_ is predicted to bind the CD44 residues with its K_296_, F_300_, E_293_, E_294_ and K_292_ residues, respectively. (C) Refined predicted model of OPN_217–230_ peptide bound to ordered conformation of CD44. Prediction of OPN_217–230_ docking to the ordered conformation of extracellular portion of CD44 hyaloronan binding domain (PDB: 1POZ). OPN_217–230_ E_226_ and S_228_ residue interactions with CD44 N_164_ were labelled as contact points in CABS‐dock runs. The predicted model placed peptide between the α1 helix and flexible loop might stabilise the CD44 ordered conformation.

**Figure:**
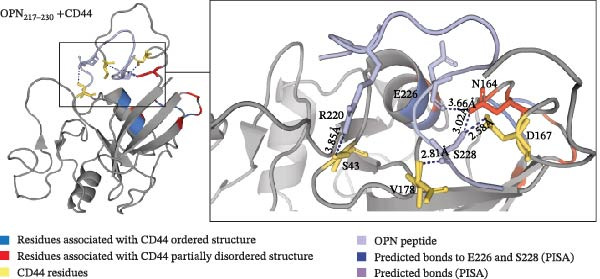
(A)

**Figure:**
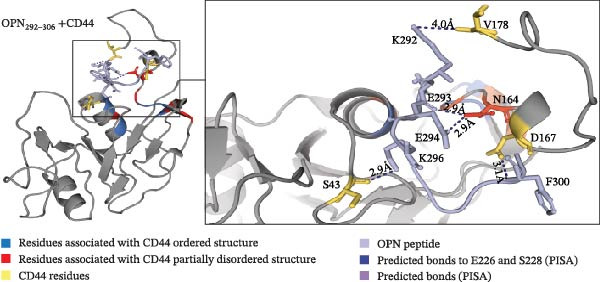
(B)

**Figure:**
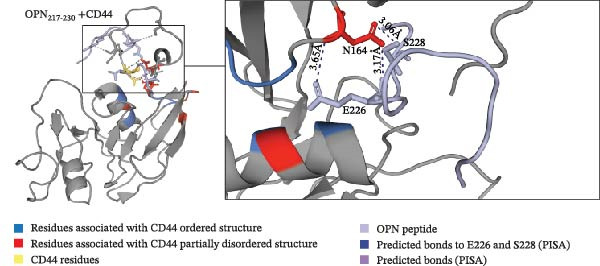
(C)

Since neither OPN_217–230_ nor OPN_292–306_ peptides reduced the PBL adhesion in presence of hyaluronan (Figure 2E), we hypothesised that they were not binding CD44 in the hyaluronan‐specific binding site and hence, labelled that binding site as ‘unlikely to bind‘ regions in the CABS‐dock prediction parameters. The predicted structure outcomes identified CD44 residues S_43_, D_167_, N_164_ and V_178_ as common residues binding to both OPN‐derived peptides, suggesting these residues could represent regions of interest when identifying the binding sites of the OPN peptides (Figure 3A, B). The N_164_ residue of CD44 has been shown to form stabilising hydrogen bonds with N_162_ in the partially disordered conformation of CD44 [12], thereby suggesting that peptide interactions with the CD44 N_164_ residue could have significance in mediating an OPN peptide‐mediated conformational change of CD44. According to our first peptide docking prediction, the CD44 N_164_ residue was predicted to bind the E_226_ and S_228_ residues of the OPN_217–230,_ and E_293_ and E_294_ residues of the OPN_292–306_ peptide (Figure 3A, B). As proof of principle, we focused on the former OPN‐derived peptide and refined the docking input in CABS‐dock by selecting contact sites between the CD44 N_164_ and the E_226_ and S_228_ residues of the OPN_217–230_ peptide. This pivoted the OPN_217–230_ peptide in between the CD44 flexible C‐terminal tail and α1‐helix, an association previously shown to stabilise the ordered conformation of CD44 [12] (Figure 3C).

Peptide‐protein docking predictions described here demonstrate a hypothesis of the molecular interaction between the investigated OPN‐derived peptides and CD44. We theorise that the OPN‐derived peptide could interact with CD44 to disrupt residues stabilising the ordered conformation via the E_226_ and S_228_ OPN residues, thereby promoting the conformational change to the partially disordered state, and in turn initiating CD44‐mediated migration (Figure 3C).

### 2.4. The E_226_ and S_228_ Residues of the Proteasome‐Generated OPN_217–230_ Peptide Impact Cell Migration

This initial peptide docking prediction suggested that the E_226_ and S_228_ residues of the OPN_217–230_ peptide could play a key role in activating CD44 (perhaps by regulating its conformation dynamics via its N_164_ residue). To investigate this hypothesis, we tested the impact of an alanine (A) substitution on one or both of the OPN_217–230_ E_226_ and S_228_ residues and repeated the CABS‐dock prediction as described earlier. Neither A‐mutated peptides, nor an OPN_217–230_ peptide with an inverse sequence, were predicted to be in close proximity to the N_164_ residue of CD44, suggesting mutations to the E_226_ and S_228_ could impact the proposed binding sites of the OPN‐derived peptides to CD44 (Figure S6A–D).

To assess the impact on cell migration of the OPN_217–230_ residues identified in our preliminary docking predictions, we synthesised variations of the wild type OPN_217–230_ peptide upon E_226_A, S_228_A and E_226_A/S_228_A substitutions as well as the inverted sequence. We then repeated the cell migration assay upon HUVECs stimulation with the wild type and mutated peptides. In contrast to the wild type OPN_217–230_ peptide, neither mutant OPN_217–230_ peptides impacted cell migration when used at 50 and 500 nM concentrations (Figure 4A). We performed the same experiment using human PBLs and reiterated observations made with HUVECs (Figure 4B). Therefore, the in cellula experiments supported the hypothesis that the E_226_ and S_228_ residues of sOPN‐FL could be involved in the promotion of cell migration.

Figure 4HUVEC and PBL migration was impaired in the presence of mutated OPN_217–230_ peptide. (A) HUVEC migration upon treatment with OPN_217–230_ or its E226A and S228A variants at either 50 nM (left graph) or 500 nM (right graph) concentration. (B) Human PBL migration upon treatment with either OPN_217–230_ or its mutated variants, or a peptide with the inverse OPN_217–230_ sequence. In (A, B), values are a percentage of treated vs. untreated cells that migrated after 20 h and are the mean and the SD of independent experiments (A: *n* = 9, left graph; *n* = 10–11, right graph; B: *n* = 9–16). Dots represent the single experiment values. Cell migration was measured in the Boyden chamber migration assay. Normality was determined using the Shapiro–Wilk test. Statistically significant difference between groups was tested by applying a Kruskal–Wallis test. Only significant *p*‐values are shown.

**Figure:**
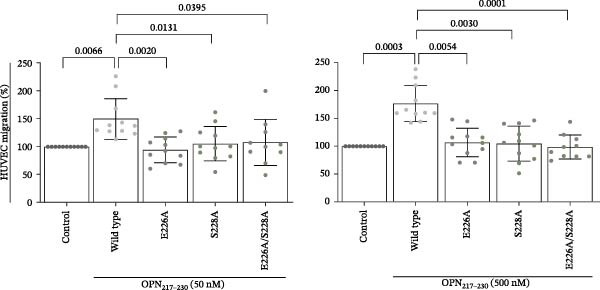
(A)

**Figure:**
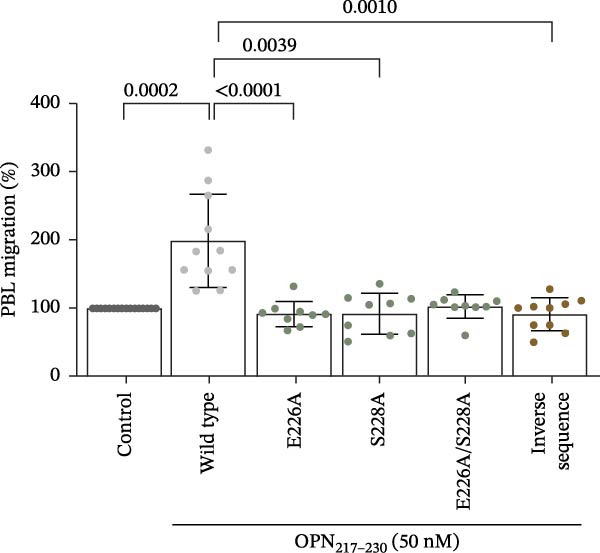
(B)

## 3. Discussion

The breakthrough study of Merbl et al. [44] put a spotlight on the role of the proteasome as a generator of peptides with functions beyond HLA‐I antigen presentation. It is hard to believe that the production of peptides by proteasomes and their release in the extracellular space is a cell autonomous defence strategy developed only as a mechanism enhanced by bacterial infection. Proteasomes are likely the most important proteases in human cells, whose activity is multi‐functional and vital to cellular maintenance. They proteolytically process a barrage of proteins, including regulators of receptor activity and other molecules located in the cell membrane. The release of peptides derived from these proteins—for example, OPNs—in the extracellular space, perhaps through pathways similar to those (still unknown) used for the anti‐bacterial peptides, could be a way cells perform autocrine/paracrine regulation. However, this might not be the only mechanism for immunologically active peptides derived from OPN to be released into the extracellular space and hence be able to modulate the activity of cell receptors such as CD44. Indeed, both proteasome and OPN have been identified in EVs, and their presence has been associated to several pathlogical conditions [18, 25–35, 55, 56]. 20S proteasomes have been shown to be active inside EVs [30, 57, 58]. We could speculate that EVs could envelop OPN‐FL leading to processing by 20S proteasomes. Consequent release of the EV contents—including proteasome‐generated OPN‐derived peptides, could be an alternative mechanism for their release into the extracellular space. In a complex extracellular environment, for example during inflammatory events, the OPN‐derived peptide half‐life and its ability to activate immunologically relevant receptors (e.g., CD44) are yet to be determined.

Although we investigated the role of OPN‐derived peptides in the stimulation of cell migration when they are in the extracellular space, these peptides might be generated by several endoproteases other than 20S proteasome, that are present both inside and outside the cells. The processing of sOPN‐FL by matrix metalloproteinases and thrombin producing OPN‐derived protein has been already described, although in those cases the cleavage products are 10 fold longer than the peptides described here and they are often the outcome of a single cleavage [1, 16]. The OPN‐derived peptides described here and in previous studies [18, 19], similarly to those described by Merbl et al. [44], have a length of 10–20 residues, which is in the range of the canonical and spliced peptides produced by proteasomes [24, 48–51] and is shorter than the average peptide product length of many endoproteases. If the presence of proteasome‐generated peptides with immunological regulatory function in the extracellular space was a mechanism that goes beyond OPN, it would be interesting to understand how this mechanism evolved.

### 3.1. Data Limitations and Perspectives

Although the study proposes a novel (potentially general) immunological regulatory mechanism of cell migration, the data has several limitations that could be addressed in future research. The first is the lack of physiological conditions demonstrating that the OPN peptides produced by proteasomes can affect cell migration when the cell producing/secreting the peptides and/or the protease are adjacent to the cells that migrate. We also cannot exclude that in physiological conditions, the peptides of interest can be generated by proteases other than 20S proteasome, trypsin, and chymotrypsin. Although our ex vivo experiments presented here and in previous studies [18, 19] consist of cell migration stimulated by synthetic peptides identified in in vitro digestions with proteasomes, we did not demonstrate that cells can secrete these peptides in a concentration sufficient for stimulating cell migration in tissues. Two key issues in providing the proper controls to these assays also include the inability to completely inhibit proteasomes in cells without causing apoptosis, and the unknown origin of extracellular sOPN and proteasomes detected in bodily fluids. They could derive from different cells and it is unclear where the OPN processing could occur, whether intracellularly then released, or in the extracellular space where proteasomes have been shown to be active [18, 29, 31, 59]. We here speculate that proteasomes process OPNs in EVs and/or in the cytoplasm and then peptides are secreted as shown for anti‐bacterial peptides [44]. In both cases, including anti‐bacterial peptides, the secretion mechanism is unknown.

The last limitation of this study is the lack of validation of the CD44‐peptide docking prediction, which may not fully encapsulate the binding dynamics of CD44 as CABS‐dock requires the input single structural models rather than NMR ensembles. The NMR ensembles used in this study could impact the residues that CABS‐dock determines are in range to form a stable bond differently to that of a crystallised model. The prediction of the OPN residues E_226_ and S_228_ as potential key player in the peptide docking is apparently supported by our in cellula experiments (Figure 4) although these residues may bind other sites than the N_164_ residue of CD44. To validate this hypothesis, techniques such as peptide‐protein cross‐linking MS and molecular dynamics stimulations could be employed. However, detecting specific and potentially transient peptide‐CD44 interactions may prove difficult and the transmembrane nature of CD44 could present technical challenges for such analyses.

## 4. Materials and Methods

### 4.1. Protein Purification

20S proteasomes used for the digestion of sOPN‐N and sOPN‐C_b_ were purified from peripheral blood as previously described [60] and the purity of the preparation has been shown [61]. 20S proteasomes used for the digestion of sOPN‐FL and sOPN‐C_a_ were purified from HeLa cells as described elsewhere [62]. The purity of the 20S proteasome preparation obtained with this method was investigated elsewhere [24] and is comparable to other purification methods [61, 63].

His‐tagged (C‐terminal) sOPN‐FL and sOPNC_a_ sequences were cloned into pGEX expression vectors (Genscript, UK) and transformed into Rosetta BL21 competent cells (Invitrogen) following the manufacturer’s protocol. Transformed bacterial colonies were cultured overnight in 5 mL of LB broth supplemented with 100 μg/mL ampicillin. Overnight cultures were then cultured in 1 L of ZYM 5052 (Media preparation STP, Francis Crick Institute) supplemented with 100 μg/mL ampicillin for 24 h at 18°C at 225 rpm and centrifuged at 4000 rpm for 8 min the following day. Cell pellets were lysed with 100 mL of lysis buffer (50 mM Tris pH8, 300 mM NaCl, 2 mM MgCL2 + 1 mM DTT + 1 tablet per 50 mL cOmplete Protease Inhibitor Cocktail (Roche), 10 μg/mL of lysozyme (Thermo Fisher Scientific) and 10 μL of benzonase (1 mg/mL in‐house, Francis Crick Institute) until completely homgenised. Lysed pellets were incubated on a roller for 30 min at 4°C. Lysed pellets were then sonicated at an amplitude of 40% for three cycles of 1 s on/1 s off for 1 min, followed by a 1 min rest between sonications. Samples were then ultracentrifuged at 20,000 rpm at 4°C for 40 min and in the meantime 1.5 mL of Cytiva Ni Sepharose high‐performance beads were washed, pelleted and recovered in water three times. This was repeated again in wash buffer (Tris pH8, 300 mM NaCl, 2 mM MgCl2, 15 mM imidazole 1 mM DTT) to equilibrate the resin. Following ultracentrifugation, the clear cell lysate was recovered and incubated with the equilibrated resin on a roller at 4°C for 2 h. Resin beads were then recovered and washed in 10 mL of wash buffer 4 times to remove unspecific binding. The desired His‐tagged protein was then washed and eluted in 4 mL elution buffer (Tris pH 8, 2 mM MgCl_2_, 100 mM NaCl, 1 mM DTT, 250 mM imidazole). Eluted samples were loaded onto washed 10 kDa Millipore Amicon Ultra Centrifugal Filters and centrifuged at 3500 rpm for 20 min to remove contaminants and concentrate the protein. All aliquots collected were ran on 4%–20% BioRad Gels as per manufacture‘s instructions. 5 μL of BioRad Precision Plus Kaleidoscope ladder was loaded to identify the molecular weight of protein bands.

Recombinant sOPN‐N and sOPN‐C_b_ were purified as described elsewhere [18].

### 4.2. In Vitro Digestion of OPN Substrates

For in vitro digestions using trypsin or chymotrypsin, 2 μg of concentrated protein was resuspended in 1% Waters RapiGest SF Surfactant and incubated for 30 min at room temperature before addition of 50 mM DTT in 25 mM ABC for 1 h at 37°C. 10 μL of IAA was added for another 1 h at 37°C. For the tryptic digest, 1 μL of trypsin (Promega) and 69 μL of 25 mM ABC buffer was incubated with the protein overnight at 37°C. For the chymotryptic digest, 2.5 μL of chymotrypsin (Promega) and 4 μL of CaCl_2_ and 63.5 μL of 25 mM ABC buffer was incubated with the proteins overnight at 37°C. 20 μL of 5% TFA was incubated with each sample at 37°C for 2 h followed by centrifugation at 13,000 rpm for 30 min. All prepared samples were concentrated via Speed‐vac.

For the in vitro digestions of OPNs by the 20S proteasome, OPNs (5 μM) were incubated over time (0–24 h) as described elsewhere [24]. Kinetics digestions with sOPN‐FL and sOPN‐C_a_ were carried out using proteasome purified from HeLa cells, whereas kinetics digestions with sOPN‐N and sOPN‐C_b_ were carried out using proteasome purified from peripheral blood [24]. All digestion time points were performed with two biological replicates for each substrate. Substrate degradation by 20S proteasomes was confirmed by Western blot as shown elsewhere [18].

### 4.3. MS Measurement and Peptide Product Analysis

In vitro digestions of sOPN‐N and sOPN‐C_b_ by 20S proteasomes were measured in duplicates (technical replicates) by Thermo Scientific Q Exactive HF‐X mass spectrometers. Briefly, during MS measurements, samples were injected using an Ultimate 3000 RSLC nano pump (both from Thermo Fisher Scientific). Peptides were loaded and separated by a nanoflow HPLC (RSLC Ultimate 3000) on a C18n column (30 cm length, 75 mm internal diameter). Peptides were eluted with a linear gradient of 5%–55% buffer B (80% ACN, 0.1% formic acid) at a flow rate of 300 nL/min for 88 min at 50°C. MS data collection was done with Thermo Xcalibur Instrument Setup v. 4.0.27.19 and Thermo Q Exactive HF Peripheral Devices v. 2.8 SP1 Build 2806 (for Q Exactive HF‐X). To acquire MS data in a Data Dependent Acquisition mode, Top 20 precursor ions were used. We acquired one full‐scan MS spectrum at a resolution of 60,000 with a normalised automatic gain control (AGC) target value of 1,000,000 and a scan range of 300–1600 m/z. The MS2 fragmentation was conducted using HCD collision energy (35%) with an orbitrap resolution of 15,000. The AGC target value was set up at 100,000 with a max injection time of 128 ms. A dynamic exclusion of 30 s and 1–6 included charged states were defined within this method. Recalibrated tandem mass spectra were matched using MSFragger v3.7.0 with a 6 ppm tolerance on precursor masses. Mass tolerance of fragment ions was set at 0.02 Da.

In vitro digestions of sOPN‐FL and sOPN‐C_a_ by 20S proteasomes, trypsin, and chymotrypsin were measured in duplicates (technical replicates) by Thermo Scientific Orbitrap Fusion mass spectrometers. Samples were injected using an Ultimate 3000 RSLC nano pump and loaded and separated by a nanoflow HPLC (RSLC Ultimate 3000) on a C18n column (30 cm length, 75 mm internal diameter). For 20S proteasome digestions, sOPN‐FL and sOPN‐C_a_ were eluted with a linear gradient of 5%–55% buffer B (80% ACN, 0.1% formic acid) at a flow rate of 300 nL/min for 88 min at 50°C. To acquire MS data in a Data Dependent Acquisition mode, Top 20 precursor ions were used. We acquired one full‐scan MS spectrum at a resolution of 120,000 with a normalised an AGC target value of 1,000,000 and a scan range of 300–1600 m/z. The MS2 fragmentation was conducted using HCD collision energy (35%) with an orbitrap resolution of 30,000. The AGC target value was set up at 100,000 with a max injection time set to dynamic. A dynamic exclusion of 30 s and 1–7 included charged states were defined within this method. Recalibrated tandem mass spectra were matched using MSFragger v3.7.0 with a 5 ppm tolerance on precursor masses. Mass tolerance of fragment ions was set at 0.02 Da.

For trypsin and chymotrypsin digestions, sOPN‐FL and sOPN‐C_a_ were eluted with a linear gradient of 5%–55% buffer B at a flow rate of 300 nL/min for 63 min at 50°C. To acquire MS data in Data Dependent Acquisition mode, a cycle time of 3 s was used. We acquired one full‐scan MS spectrum at a resolution of 60,000 with a normalised AGC target value of 400,000 and a scan range of 300–1500 m/z. The MS2 fragmentation was using HCD collision energy (35%) with an orbitrap resolution of 15,000. The AGC target value was set up at 50,000 with a max injection time of 40 ms. A dynamic exclusion of 30 s and either 1–7 or 2–7 included charge states for trypsin or chymotrypsin was used, respectively.

Tryptic and chymotryptic digestion of individual substrates sOPN‐FL, sOPN‐C_a,_ and 20S proteasome alone were analysed on PEAKS X+v10.5. As previously described [24], human and host proteome were used as the search database to generate contamination databases. Contaminant databases were then implemented with raw MS data from substrate 20S proteasome digestion for inSPIRE‐1.5—aSPIRE, as previously described [24].

### 4.4. Cell Culture

HUVECs were isolated from human umbilical veins via trypsin treatment (1%) and cultured in M199 medium (Sigma–Aldrich) with the addition of 20% FBS (Invitrogen, Burlington, ON, Canada) and 100 U/mL penicillin, 100 mg/mL streptomycin (Invitrogen), 5 UI/mL heparin (Sigma–Aldrich), 12 mg/mL bovine brain extract, and 200 mM glutamine (Hyclone Laboratories). HUVECs were grown to confluence in flasks and used at the second to fifth passage. 2 ^∗^10^3^ HUVECs were used for migration assay and cultured in M200 medium (GIBCO) with 100 U/mL penicillin, 100 mg/mL streptomycin and 200 mM glutamine.

Peripheral blood mononuclear cells were separated from buffy coat, provided by the local Blood Transfusion Service of Torino, Italy, with the Ficoll‐Hypaque reagent (Limpholyte‐H, Cedarlane Laboratories) by density‐gradient centrifugation. PBLs were obtained from peripheral blood mononuclear cells cultured in RPMI 1640 (Sigma–Aldrich) supplemented with 10% FBS, 200 mM L‐glutamine, 100 U/mL penicillin, 100 mg/mL streptomycin, and left for 2 h to remove the adherent monocytes. The cell purity was checked by immunophenotypic analysis and was higher than 98%. 5 ^∗^10^4^ PBLs were used for migration assay and cultured in medium X‐vivo 20 (Lonza), with 100 U/mL penicillin, 100 mg/mL streptomycin and 200 mM glutamine.

The use of primary human cells was approved by the Ethics Committee of the ‘‘Presidio Ospedaliero Martini” of Torino (protocol 263‐07/NF and SanPlac/16) and conducted in accordance with the Declaration of Helsinki. Written informed consent was obtained from all donors.

### 4.5. Cell Migration Assay

In the Boyden chamber (BD Biosciences) migration assay, cells were plated onto the apical side of 50 µg/mL Matrigel‐coated filters (5 µm pore size; Neuro Probe; BIOMAP snc) in M200 serum‐free medium for HUVECs or X‐vivo serum‐free medium for PBLs. 10 µg/mL OPN‐C or different concentrations of OPN peptides were placed in the basolateral chamber. 10 ng/mL vascular endothelial growth factor‐α (VEGF‐α; Sigma–Aldrich) or 200 ng/mL recombinant human RANTES (rh RANTES/CCL5; ImmunoTools GmbH) are placed as reference chemoattractant for the HUVECs or the PBLs. 20S proteasomes were pre‐incubated with sOPN‐C (with ratio ng proteasome: ng OPNs = 20:1) for 2 h at 37°C under 5% CO_2_ and subsequently placed in the basolateral chamber. The chamber was incubated at 37°C under 5% CO_2_. After 20 h, the cells on the apical side were wiped off with Q‐tips. The cells on the bottom of the filter were stained with crystal violet (HUVECs) or eosin Y and thiazine (PBLs) and all counted with an reverse microscope (magnification x40). Data are shown as percentage of the treated cells migration *vs* the control migration measured for untreated cells. Control migration (mean ± SD) 87 ± 7 cells for HUVECs and 105 ± 9 cells for PBLs.

### 4.6. Adhesion Assay

For the adhesion assay to hyaluronan (HA), 24‐microwell plates (Costar, Corning Inc., NY, USA) were coated overnight at 37°C with 2.5 mg/mL hyaluronic acid sodium salt (Abcam). Coated wells were then incubated with 1% heat‐denatured BSA in phosphate‐buffered saline for 30 min at 37°C. After 30 min, BSA was aspired and, for each condition, 1 × 10^5^ PBLs were resuspended in X‐VIVO medium (Lonza) were seeded on pre‐coated wells and pre‐incubated with 1 µg/mL α‐CD44 (Ancell) for 30 min at 37°C. PMA 1 ng/mL was used as the control stimulus. Thereafter, cells were treated with synthetic OPN_217–230_ (50 nM) for 3 h at 37°C. Unattached lymphocytes were washed away, and the number of adherent cells was evaluated by the Image Pro Plus Software for micro‐imaging (Media Cybernetics, version 5.0, Bethesda, MD).

### 4.7. Prediction of Peptide Docking

Prediction of OPN‐CD44 binding sites was conducted using the protein‐peptide docking prediction tool, CABS‐dock (available at: https://biocomp.chem.uw.edu.pl/CABSdock). The NMR structure ensemble representing the ordered conformation (PDB: 1POZ) of CD44 was used to predict docking. The first NMR structure amongst the 20 NMR structures was selected as it allowed the flexibility of the tail as described by Takeda et al. [54]. OPN peptide sequences (OPN‐FL_217–230_: WDSRGKDSYETSQL and OPN‐FL_292–306_: KEEDKHLKFRISHEL) were used to predict peptide‐CD44 binding. CABS‐dock runs were customised to exclude CD44 residues found to bind hyaluronan (HA) (K38, R41, Y42 K68, R78, Y79, N100, N101, Y101) [64], labelled as ‘unlikely to bind regions’. CABs‐dock results generated 10 representative models, ranked on the binding energy of the model. All predicted models were assessed for binding residues using PDBePISA (Protein, Interfaces, Structures and Assembilies) which identified hydrogen binding and salt bridges between the peptide and protein. PISA results generated, were assessed to remove models that predicted to bind CD44 HA‐binding residues and the highest‐ranking model was selected. Residues found to bind CD44 were compared to those found in literature to effect the conformational structure of CD44 and refinements were made to the CABS‐dock input, selecting any residues of importance as contact points. Peptide‐protein docking models were visualised in PyMol CD44 and residues found to bind both OPN_217–230_ and OPN_292–306_ peptides were identified and labelled. Binding residues found were compared to those found in literature to contribute to the conformational change of CD44 [12]. Three mutant peptides were generated; OPN_217–292_ E226A (WDSRGKDSYATSQL), OPN_217–292_ S228A (WDSRGKDSYETAQL) and OPN_217–292_ E10A/S12A (WDSRGKDSYATAQL). Docking predictions for mutated peptides were assessed following the strategy adopted for the wild type peptides. Additionally, CD44‐OPN binding was validated using the OPN_217–230_ inverse sequence (TEYSDKGRSDW), mutant and inverse peptide docking predictions, which were compared to the wild type OPN_217–292._ Notable CD44 binding OPN residues were selected for in vitro mutation experiments.

### 4.8. Statistical Analysis

Using R Studio and Graphpad Prism, data have been tested for normality distribution and homoscedasticity using the Shapiro–Wilk normality test. Parametric and non‐parametric statistical tests are described in each figure caption. Only *p*‐value <0.05 has been considered statistically significant and reported with its value.

## Author Contributions

Hindh Imad, Kathryn E. Strange, Michele Mishto and Juliane Liepe developed the project and wrote the manuscript, which was proofread by all other authors. Hindh Imad performed peptide docking and structural biology analyses under the supervision of Timothy James Nott, Kathryn E. Strange, Michele Mishto and Juliane Liepe. Hindh Imad and Kathryn E. Strange synthesised the sOPN‐FL and sOPN‐C_a_ substrates and performed the data analysis, which was supervised by Michele Mishto, Juliane Liepe and Kathryn E. Strange. Wai Tuck Soh and Sophie Siddons handled the MS measurements and processed the resulting data under the supervision of Henning Urlaub. Casimiro Luca Gigliotti prepared the ex vivo PBL samples. Chiara Dianzani performed the ex vivo assays in collaboration with Umberto Dianzani. Annalisa Chiocchetti produced the sOPN‐C_b_ and sOPN‐N recombinant proteins.

## Funding

This work was financed in part by: (i) CRUK City of London Centre (CoL) Award [CTRQQR‐2021/100004] and Blood Cancer UK [Ref. 22009] to MM and SK; (ii) ERC‐StG 945528 IMAP to JL; (iii) the Max Planck Society to JL and HU. WTS was supported by the European Union’s Framework Programme for Research and Innovation Horizon Europe (2021−2027) under the Marie Skłodowska‐Curie Grant Agreement No. 101065466. This work was supported by the Francis Crick Institute which receives its core funding from Cancer Research UK (CC0102), the UK Medical Research Council (CC0102), and the Wellcome Trust (CC0102). For the purpose of Open Access, the author has applied a CC BY public copyright licence to any Author Accepted Manuscript version arising from this.

## Conflicts of Interest

Michele Mishto consults for G.S.K. The other authors declare no conflicts of interest.

## Supporting Information

Additional supporting information can be found online in the Supporting Information section.

## Supporting information


**Supporting Information** Table S1: Sequence of the recombinant OPN proteins digested by 20S proteasomes. Table S2: OPN_217-230_ and OPN_292-306_ peptides and their N‐ and C‐terminally extended versions identified in the in vitro digestions of sOPN‐FL, sOPN‐Ca and sOPN‐Cb by 20S proteasomes. Table S3: In vitro digestion of OPN‐FL and OPN‐C with trypsin and chymotrypsin. Figure S1: Recombinant OPN proteins digested by 20S proteasomes. Figure S2: PSM features and hotspot regions of sOPN‐FL in vitro digestions with 20S proteasomes. Figure S3: MS2 spectra and peptide generation kinetics of the OPN_217-230_ peptide identified in the in vitro digestions of sOPN‐FL and sOPN‐Ca by 20S proteasomes. Figure S4: Inhibition of unstimulated migration of HUVECs. Figure S5: Hypothesis of activation of the hyaluronan‐mediated cell migration by OPN‐derived peptides. Figure S6: Prediction of the binding of OPN_217-230_ mutated peptides to CD44.

## Data Availability

The MS files of the sOPN‐FL, sOPN‐C_a_, sOPN‐C_b_ and sOPN‐N digestions by 20S proteasomes as well as the inSPIRE 1.5 output files are available at the MassIVE online repository with the dataset identifier MSV000098438 and MSV000100096.
