# Supplementary material for: OPN‐Derived Peptides Generated by Proteasomes Can Promote Cell Migration via CD44 Activation
Source: J Immunol Res. 2026 Feb 25;2026:2726052. doi: 10.1155/jimr/2726052 (PMC13140175; doi:10.1155/jimr/2726052)
Supplement: Supplementary file 1 — Supporting Information Table S1: Sequence of the recombinant OPN proteins digested by 20S proteasomes. Table S2: OPN217-230 and OPN292-306 peptides and their N‐ and C‐terminally extended versions identified in the in vitro digestions of sOPN‐FL, sOPN‐Ca and sOPN‐Cb by 20S proteasomes. Table S3: In vitro digestion of OPN‐FL and OPN‐C with trypsin and chymotrypsin. Figure S1: Recombinant OPN proteins digested by 20S proteasomes. Figure S2: PSM features and hotspot regions of sOPN‐FL in vitro digestions with 20S proteasomes. Figure S3: MS2 spectra and peptide generation kinetics of the OPN217-230 peptide identified in the in vitro digestions of sOPN‐FL and sOPN‐Ca by 20S proteasomes. Figure S4: Inhibition of unstimulated migration of HUVECs. Figure S5: Hypothesis of activation of the hyaluronan‐mediated cell migration by OPN‐derived peptides. Figure S6: Prediction of the binding of OPN217-230 mutated peptides to CD44. [file JIMR-2026-2726052-s001.pdf]

OPN-derived peptides generated by proteasomes can promote cell migration via CD44 activation

To whom the correspondence should be addressed: michele.mishto@kcl.ac.uk; jliepe@mpinat.mpg.de.

Supplementary Table 1	Sequence of the recombinant OPN proteins digested by 20S proteasomes
Supplementary Table 2	$OPN_{217\text{-}230}$ and $OPN_{292\text{-}306}$ peptides and their N- and C-terminally extended versions identified in the *in vitro* digestions of sOPN-FL, sOPN-C_a and sOPN-C_b by 20S proteasomes.
Supplementary Table 3	*In vitro* digestion of OPN-FL and OPN-C with trypsin and chymotrypsin
Supplementary Figure 1	Recombinant OPN proteins digested by 20S proteasomes
Supplementary Figure 2	PSM features and hotspot regions of sOPN-FL *in vitro* digestions with 20S proteasomes
Supplementary Figure 3	MS2 spectra and peptide generation kinetics of the $OPN_{217\text{-}230}$ peptide identified in the *in vitro* digestions of sOPN-FL and sOPN-C_a by 20S proteasomes
Supplementary Figure 4	Inhibition of unstimulated migration of HUVECs
Supplementary Figure 5	Hypothesis of activation of the hyaluronan-mediated cell migration by OPN-derived peptides
Supplementary Figure 6	Prediction of the binding of $OPN_{217\text{-}230}$ mutated peptides to CD44

Protein name	Position within sOPN-FL	Sequence
**sOPN-FL**	17-314	**HHHHHH**DYDIPTTENLYFQGAHGSIPVKQADSGSSEEKQLYNKYPDAVA<br>TWLNPDPSQKQNLLAPQNAVSSEETNDFKQETLPSKSNESHDHMDDMD<br>DEDDDDHVDSQDSIDSNDSDDVDDTDDSHQSDESHHSDESDELVTDFP<br>TDLPATEVFTPVVPTVDTYDGRGDSVVYGLRSKSKKFRRPDIQYPDATDE<br>DITSHMESEELNGAYKAIPVAQDLNAPSDWDSRGKDSYETSQLDDQSAE<br>THSHKQSRLYKRKANDESNEHSDVIDSQELSKVSREFHSHEFHSHEDML<br>VVDPKSKEEDKHLKFRISHELDSASSEVN
**sOPN-N**	17-168	IPVKQADSGSSEEKQLYNKYPDAVATWLNPDPSQKQNLLAPQNAVSSEE<br>TNDFKQETLPSKSNESHDHMDDMDDEDDDDHVDSQDSIDSNDSDDVDD<br>TDDSHQSDESHHSDESDELVTDFPTDLPATEVFTPVVPTVDTYDGRGDS<br>VVYGLR**HHHHHH**
**sOPN-C_a**	169-314	**HHHHHH**DYDIPTTENLYFQGAHGSSKSKKFRRPDIQYPDATDEDITSHME<br>SEELNGAYKAIPVAQDLNAPSDWDSRGKDSYETSQLDDQSAETHSHKQS<br>RLYKRKANDESNEHSDVIDSQELSKVSREFHSHEFHSHEDMLVVDPKSK<br>EEDKHLKFRISHELDSASSEVN
**sOPN-C_b**	169-314	SKSKKFRRPDIQYPDATDEDITSHMESEELNGAYKAIPVAQDLNAPSDWD<br>SRGKDSYETSQLDDQSAETHSHKQSRLYKRKANDESNEHSDVIDSQELS<br>KVSREFHSHEFHSHEDMLVVDPKSKEEDKHLKFRISHELDSASSEVN**HH**<br>**HHHH**

Supplementary Table 1. Sequence of the recombinant OPN proteins digested by 20S proteasomes. In the study we digested with 20S proteasomes 4 recombinant proteins, *i.e.*, sOPN-FL, sOPN-N, sOPN-C_a, sOPN-C_b. Tags are labelled in red, RGD sequence underlined.

A)

**Substrate**	**$OPN_{217-230}$ and terminally extended versions**	**Extension**	**Spectral Angle**
sOPN-FL	**WDSRGKDSYETSQL**	None	0.82
sOPN-FL	**WDSRGKDSYETSQL**D	C-terminus	0.87
sOPN-FL	**WDSRGKDSYETSQL**DD	C-Terminus	0.89
sOPN-FL	PSD**WDSRGKDSYETSQL**D	Both	0.85
sOPN-FL	**WDSRGKDSYETSQL**DDQS	C-terminus	0.67
sOPN-FL	NAPSD**WDSRGKDSYETSQL**	N-terminus	0.81
sOPN-FL	PSD**WDSRGKDSYETSQL**DD	Both	0.89
sOPN-FL	APSD**WDSRGKDSYETSQL**DD	Both	0.76
sOPN-FL	NAPSD**WDSRGKDSYETSQL**D	Both	0.90
sOPN-Ca	**WDSRGKDSYETSQL**	None	0.84
sOPN-Ca	NAPSD**WDSRGKDSYETSQL**DD	Both	0.83
sOPN-Ca	PSD**WDSRGKDSYETSQL**D	Both	0.86
sOPN-Ca	NAPSD**WDSRGKDSYETSQL**D	Both	0.85

B)

**Substrate**	**$OPN_{292-306}$ and terminally extended versions**	**Extension**	**Spectral Angle**
sOPN-FL	**KEEDKHLKFRISHELD**	None	0.79
sOPN-FL	**KEEDKHLKFRISHELD**SA	C-terminus	0.70
sOPN-FL	PKS**KEEDKHLKFRISHELD**	N-terminus	0.72
sOPN-FL	**KEEDKHLKFRISHELD**SASSE	C-terminus	0.83
sOPN-Ca	DPKS**KEEDKHLKFRISHELD**	N-terminus	0.58
sOPN-Ca	PKS**KEEDKHLKFRISHELD**SASSE	Both	0.79
sOPN-Cb	S**KEEDKHLKFRISHELD**SA	Both	0.72
sOPN-Cb	PKS**KEEDKHLKFRISHELD**SA	Both	0.79

Supplementary Table 2. $OPN_{217-230}$ and $OPN_{292-306}$ peptides and their N- and C- terminally extended versions identified in the *in vitro* digestions of sOPN-FL, sOPN-C_a and sOPN-C_b by 20S proteasomes. (**A,B**) The list of the $OPN_{217-230}$ (**A**) and $OPN_{292-306}$ (**B**) peptides and their extensions (within a range of 5 residues) among the *in vitro* digestions of sOPN-FL, sOPN-C_a and sOPN-C_b with 20S proteasomes. The peptides were identified by MS and the analysis of the MS files by inSPIRE 1.5. The presence of terminal extensions and the spectral angles of the MS2 spectra is provided.

Protein origin	Sequence	Digestion type
sOPN-FL	GKDSYETSQLDDQSAETHSHK	Trypsin
sOPN-FL	GKDSYETSQLDDQSAETHSHKQSR	Trypsin
sOPN-FL	GKDSYETSQLDDQSAETHSHKQSR	Trypsin
sOPN-FL	KANDESNEHSDVIDSQELSK	Trypsin
sOPN-FL	KANDESNEHSDVIDSQELSK	Trypsin
sOPN-FL	ANDESNEHSDVIDSQELSK	Trypsin
sOPN-FL	GKDSYETSQLDDQSAETHSHK	Trypsin
sOPN-FL	QNLLAPQNAVSSEETNDFKQETLPSK	Trypsin
sOPN-FL	RPDIQYPDATDEDITSHMESEELNGAYK	Trypsin
sOPN-FL	DSYETSQLDDQSAETHSHK	Trypsin
sOPN-FL	ANDESNEHSDVIDSQELSK	Trypsin
sOPN-FL	AIPVAQDLNAPSDWDSR	Trypsin
sOPN-FL	ANDESNEHSDVIDSQELSK	Trypsin
sOPN-FL	YPDAVATWLNPDPSQK	Trypsin
sOPN-FL	GDSVVYGLR	Trypsin
sOPN-FL	AIPVAQDLNAPSDWDSR	Trypsin
sOPN-FL	QLYNKYPDAVATWLNPDPSQK	Trypsin
sOPN-FL	FRISHELDSASSEVN	Trypsin
sOPN-FL	QNLLAPQNAVSSEETNDFKQETLPSK	Trypsin
sOPN-FL	QNLLAPQNAVSSEETNDFKQETLPSK	Trypsin
sOPN-FL	RPDIQYPDATDEDITSHMESEELNGAYK	Trypsin
sOPN-FL	QNLLAPQNAVSSEETNDFKQETLPSK	Trypsin
sOPN-FL	DSYETSQLDDQSAETHSHK	Trypsin
sOPN-FL	QNLLAPQNAVSSEETNDFKQETLPSK	Trypsin
sOPN-FL	ISHELDSASSEVN	Trypsin
sOPN-FL	FRISHELDSASSEVN	Trypsin
sOPN-FL	ISHELDSASSEVN	Trypsin
sOPN-FL	RPDIQYPDATDEDITSHMESEELNGAYK	Trypsin
sOPN-FL	QLYNKYPDAVATWLNPDPSQK	Trypsin
sOPN-FL	RKANDESNEHSDVIDSQELSK	Trypsin
sOPN-FL	KANDESNEHSDVIDSQELSK	Trypsin
sOPN-FL	AIPVAQDLNAPSDWDSRGK	Trypsin
sOPN-FL	QNLLAPQNAVSSEETNDFK	Trypsin
sOPN-FL	AIPVAQDLNAPSDWDSRGK	Trypsin
sOPN-FL	SKEEDKHLK	Trypsin
sOPN-FL	QADSGSSEEKQLYNKYPDAVATWLNPDPSQK	Trypsin
sOPN-FL	ANDESNEHSDVIDSQELSKVSR	Trypsin
sOPN-FL	RPDIQYPDATDEDITSHMESEELNGAYK	Trypsin
sOPN-FL	QADSGSSEEKQLYNKYPDAVATWLNPDPSQK	Trypsin
sOPN-FL	KANDESNEHSDVIDSQELSKVSR	Trypsin
sOPN-FL	QADSGSSEEKQLYNKYPDAVATWLNPDPSQK	Trypsin
sOPN-FL	QLYNKYPDAVATWLNPDPSQK	Trypsin
sOPN-FL	DSRGKDSYETSQLDDQSAETHSHKQSRLY	Chymotrypsin
sOPN-FL	DSRGKDSYETSQLDDQSAETHSHKQSRLY	Chymotrypsin
sOPN-FL	RRPDIQYPDATDEDITSHMESEELNGAY	Chymotrypsin
sOPN-FL	RRPDIQYPDATDEDITSHMESEELNGAY	Chymotrypsin
sOPN-FL	FQGAHGSIPVKQADSGSSEEKQLY	Chymotrypsin
sOPN-FL	FQGAHGSIPVKQADSGSSEEKQLY	Chymotrypsin
sOPN-FL	RISHELDSASSEVN	Chymotrypsin
sOPN-FL	DSRGKDSYETSQLDDQSAETHSHKQSRLY	Chymotrypsin
sOPN-FL	KAIPVAQDLNAPSDW	Chymotrypsin
sOPN-FL	KRKANDESNEHSDVIDSQELSKVSREF	Chymotrypsin
sOPN-FL	DDQSAETHSHKQSRLY	Chymotrypsin
sOPN-FL	KRKANDESNEHSDVIDSQEL	Chymotrypsin
sOPN-FL	LNPDPSQKQNLL	Chymotrypsin
sOPN-FL	RISHELDSASSEVN	Chymotrypsin
sOPN-FL	TPVVPTVDTY	Chymotrypsin
sOPN-FL	KFRISHELDSASSEVN	Chymotrypsin
sOPN-FL	KAIPVAQDLNAPSDWDSRGKDSYETSQL	Chymotrypsin
sOPN-FL	RRPDIQYPDATDEDITSHMESEELNGAY	Chymotrypsin
sOPN-FL	KAIPVAQDLNAPSDWDSRGKDSY	Chymotrypsin
sOPN-FL	NKYPDAVATW	Chymotrypsin

sOPN-FL	FQGAHGSIPVKQADSGSSEEKQLYNKYPDAVATW	Chymotrypsin
sOPN-FL	KRKANDESNEHSDVIDSQELSKVSREF	Chymotrypsin
sOPN-FL	DSRGKDSYETSQLDDQSAETHSHKQSRL	Chymotrypsin
sOPN-FL	KRKANDESNEHSDVIDSQEL	Chymotrypsin
sOPN-FL	YFQGAHGSIPVKQADSGSSEEKQLY	Chymotrypsin
sOPN-FL	TPVVPTVDTYDGRGDSVVY	Chymotrypsin
sOPN-FL	ETSQLDDQSAETHSHKQSRLY	Chymotrypsin
sOPN-FL	DSRGKDSYETSQLDDQSAETHSHKQSRL	Chymotrypsin
sOPN-C	GKDSYETSQLDDQSAETHSHKQSR	Trypsin
sOPN-C	KANDESNEHSDVIDSQELSK	Trypsin
sOPN-C	GKDSYETSQLDDQSAETHSHK	Trypsin
sOPN-C	ANDESNEHSDVIDSQELSK	Trypsin
sOPN-C	KANDESNEHSDVIDSQELSK	Trypsin
sOPN-C	DSYETSQLDDQSAETHSHK	Trypsin
sOPN-C	RKANDESNEHSDVIDSQELSK	Trypsin
sOPN-C	ANDESNEHSDVIDSQELSK	Trypsin
sOPN-C	RPDIQYPDATDEDITSHMESEELNGAYK	Trypsin
sOPN-C	AIPVAQDLNAPSDWDSR	Trypsin
sOPN-C	FRISHELDSASSEVN	Trypsin
sOPN-C	RPDIQYPDATDEDITSHMESEELNGAYK	Trypsin
sOPN-C	FRISHELDSASSEVN	Trypsin
sOPN-C	AIPVAQDLNAPSDWDSR	Trypsin
sOPN-C	ISHELDSASSEVN	Trypsin
sOPN-C	GKDSYETSQLDDQSAETHSHK	Trypsin
sOPN-C	DSYETSQLDDQSAETHSHK	Trypsin
sOPN-C	RKANDESNEHSDVIDSQELSK	Trypsin
sOPN-C	KANDESNEHSDVIDSQELSK	Trypsin
sOPN-C	ISHELDSASSEVN	Trypsin
sOPN-C	AIPVAQDLNAPSDWDSRGK	Trypsin
sOPN-C	AIPVAQDLNAPSDWDSRGK	Trypsin
sOPN-C	SKEEDKHLK	Trypsin
sOPN-C	RPDIQYPDATDEDITSHMESEELNGAYK	Trypsin
sOPN-C	KANDESNEHSDVIDSQELSKVSR	Trypsin
sOPN-C	EFHSHEFHSHEDMLVVDPK	Trypsin
sOPN-C	RRPDIQYPDATDEDITSHMESEELNGAY	Chymotrypsin
sOPN-C	DSRGKDSYETSQLDDQSAETHSHKQSRLY	Chymotrypsin
sOPN-C	KAIPVAQDLNAPSDW	Chymotrypsin
sOPN-C	RRPDIQYPDATDEDITSHMESEELNGAY	Chymotrypsin
sOPN-C	RISHELDSASSEVN	Chymotrypsin
sOPN-C	DDQSAETHSHKQSRLY	Chymotrypsin
sOPN-C	KFRISHELDSASSEVN	Chymotrypsin
sOPN-C	KRKANDESNEHSDVIDSQELSKVSREF	Chymotrypsin
sOPN-C	KAIPVAQDLNAPSDWDSRGKDSY	Chymotrypsin
sOPN-C	RISHELDSASSEVN	Chymotrypsin
sOPN-C	KRKANDESNEHSDVIDSQELSKVSREF	Chymotrypsin
sOPN-C	DSRGKDSYETSQLDDQSAETHSHKQSRL	Chymotrypsin
sOPN-C	DSRGKDSYETSQLDDQSAETHSHKQSRLY	Chymotrypsin
sOPN-C	KAIPVAQDLNAPSDW	Chymotrypsin
sOPN-C	FQGAHGSSKSKKF	Chymotrypsin
sOPN-C	YKRKANDESNEHSDVIDSQEL	Chymotrypsin
sOPN-C	ETSQLDDQSAETHSHKQSRLY	Chymotrypsin
sOPN-C	YFQGAHGSSKSKKF	Chymotrypsin
sOPN-C	DSRGKDSYETSQLDDQSAETHSHKQSRL	Chymotrypsin
sOPN-C	KFRISHELDSASSEVN	Chymotrypsin

Supplementary Table 3. *In vitro* digestion of sOPN-FL and sOPN-C with trypsin and chymotrypsin. Peptides identified in the trypsin and chymotrypsin digestion of either OPN-FL$_{17\text{-}314}$ or OPN-C$_{169\text{-}314}$.

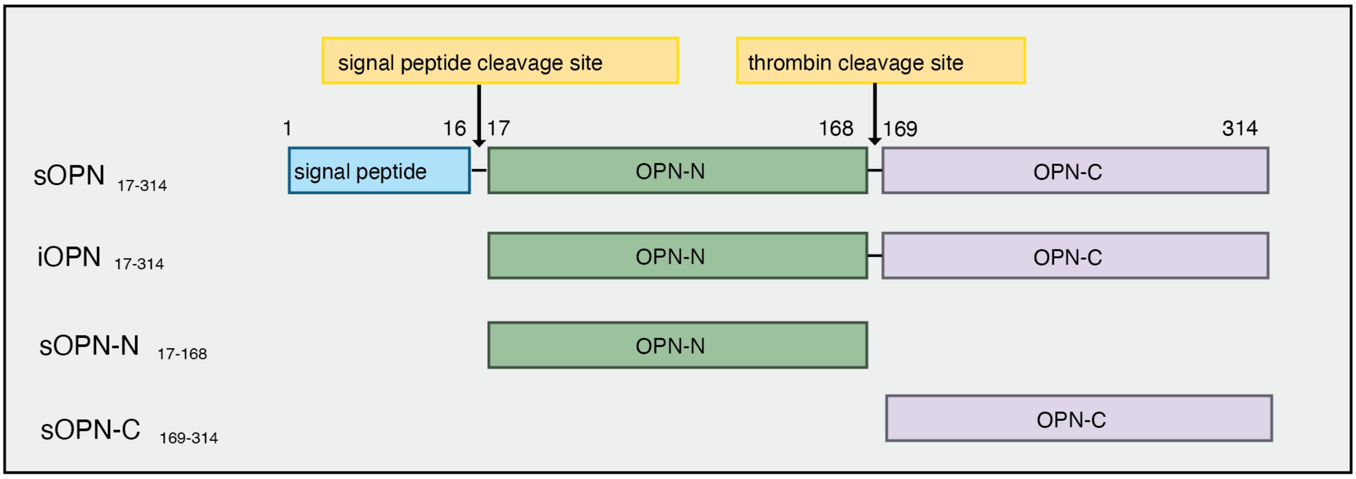

Supplementary Figure 1. Recombinant OPN proteins digested by 20S proteasomes. For the detail of the recombinant substrate sequence, see **Supplementary Table 1**.

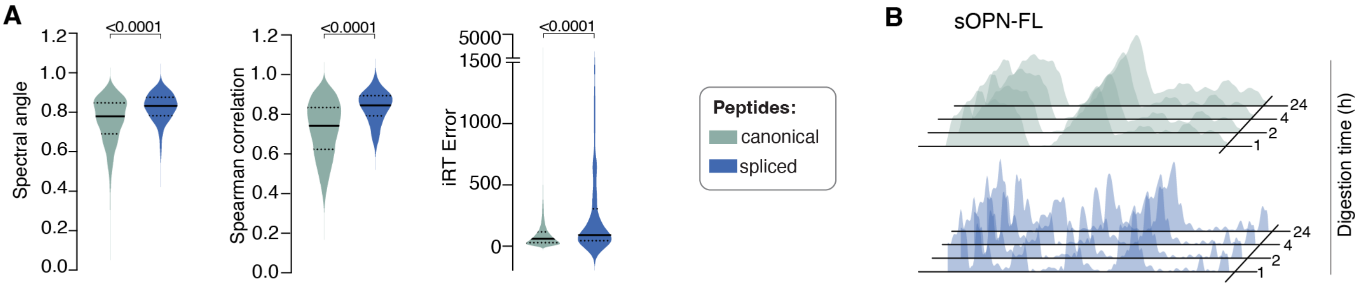

Supplementary Figure 2. PSM features and hotspot regions of sOPN-FL *in vitro* digestions with 20S proteasomes. (**A**) Spectral angle (p=7.92e-27), Spearman correlation (p= 2.68e-54) and iRT (p= 2.34e-18) values for all assigned PSMs of peptides identified in the *in vitro* digestions of sOPN-FL with 20S proteasomes and computed by applying inSPIRE 1.5 software. Statistically significant difference between groups was tested by applying non-paired two-sided Wilcoxon rank sum test with continuity correction. (**B**) Protein coverage profiles of canonical and spliced peptide products of *in vitro* digestions of sOPN-FL with 20S proteasomes over the time points of the degradation kinetics, as generated with aSPIRE software. The sOPN-FL is representative of all others. For all digestions, n = 2 biological replicates were performed per substrate, each measured twice by MS.

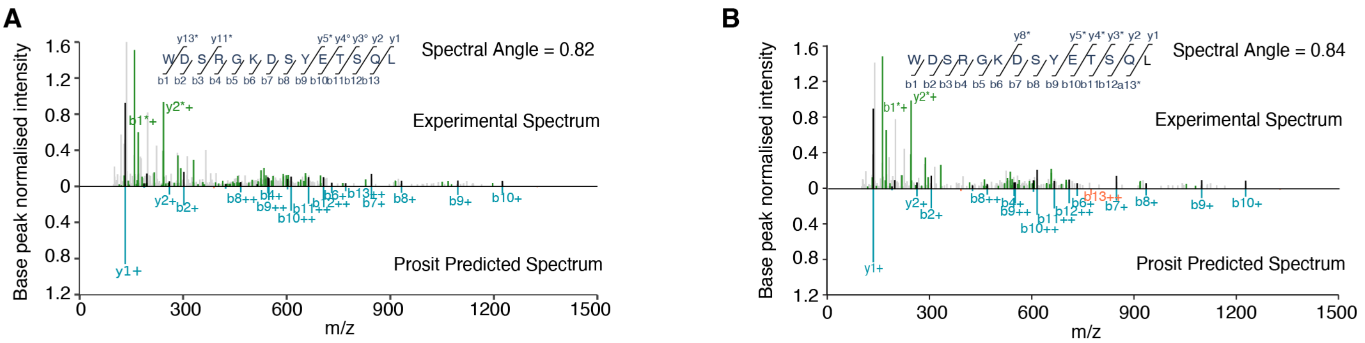

Supplementary Figure 3. MS2 spectra of the OPN$_{217\text{-}230}$ peptide identified in the *in vitro* digestions of sOPN-FL and sOPN-C$_a$ by 20S proteasomes. Example MS2 spectra of the OPN$_{217\text{-}230}$ peptide identified in the *in vitro* digestions of sOPN-FL (**A**) and sOPN-C$_a$ (**B**) by 20S proteasomes compared to the theoretical MS2 spectra predicted by Prosit. Peptides were detected as such (considering I/L redundancy) in both biological replicates performed. Plots comparing the experimental spectrum for an identified peptide on the positive y-axis against the Prosit predicted spectrum for that peptide on the negative y-axis. Comparisons are shown for the best scoring spectrum of all peptides identified at 1% FDR. Potential y-, b-, or a-ions matched between spectra are in green. Matched peaks of unknown origin are in black. Peaks not matched are in grey. Charge is shown by number $^+$ symbols. Ions' loss of water or ammonia is symbolized by ^{O} and *.

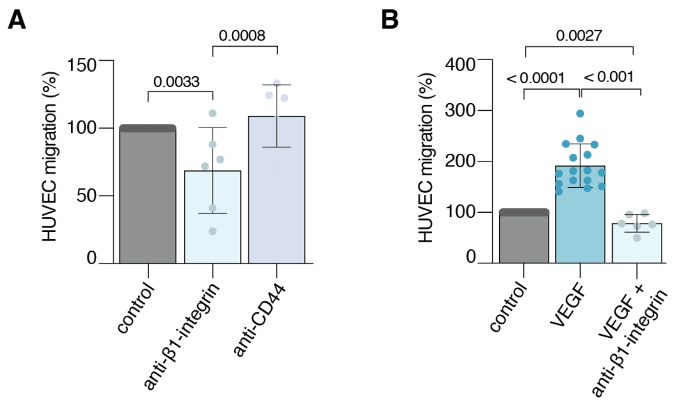

Supplementary Figure 4. Inhibition of unstimulated migration of HUVECs. (**A**) Migration of HUVECs upon treatment with antagonist antibodies blocking either integrin β1 or CD44. (**B**) Migration of HUVECs upon treatment with VEGF antagonist antibodies blocking integrin β1. In (**A-B**), values are percentage of treated vs untreated cells that migrated after 20 h and are the mean and the SD of independent experiments (A: n = 6 – 38 B: n = 16 - 38). Cell migration has been measured in the Boyden chamber migration assay. Normal distribution was tested by using the Shapiro-Wilk test and statistically significant p-values (Kurskal-Wallis test for paired samples) are shown.

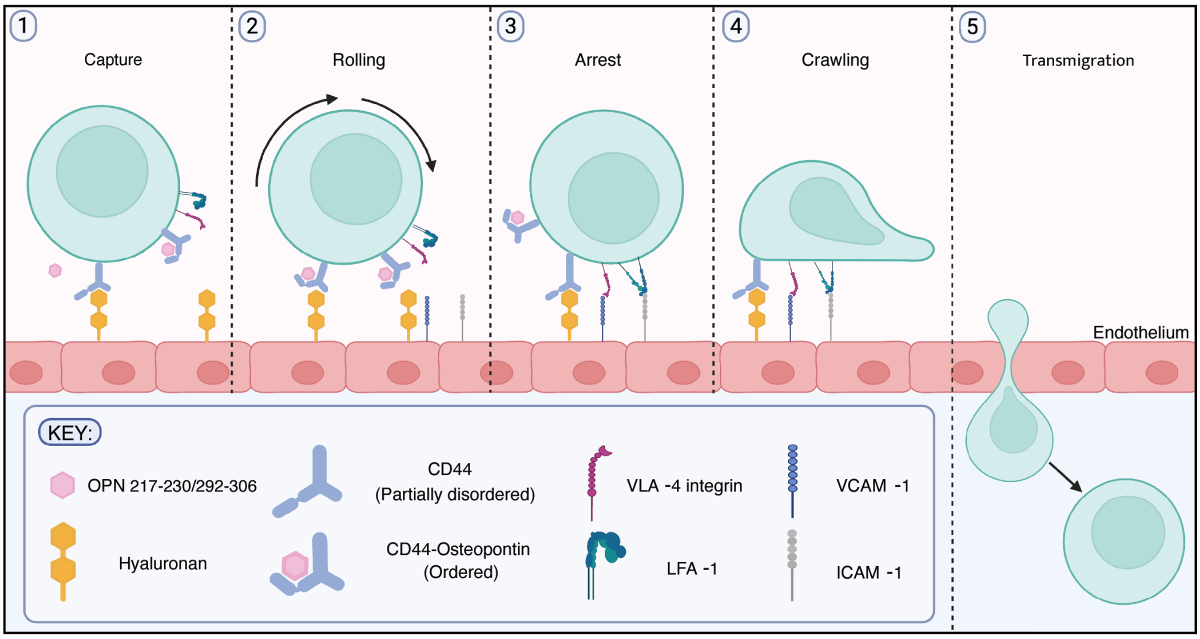

Supplementary figure 5. Hypothesis of activation of the hyaluronan-mediated cell migration by OPN-derived peptides. Schematic of the possible role of $OPN_{217\text{-}230}$ and $OPN_{292\text{-}306}$ peptides in activating different steps of the hyaluronan-mediated PBL cell migration via CD44 activation. The step of arrest (adhesion) seems to be unaffected by the binding of the $OPN_{217\text{-}230}$ and $OPN_{292\text{-}306}$ peptides in PBLs (see **Fig. 2E**).

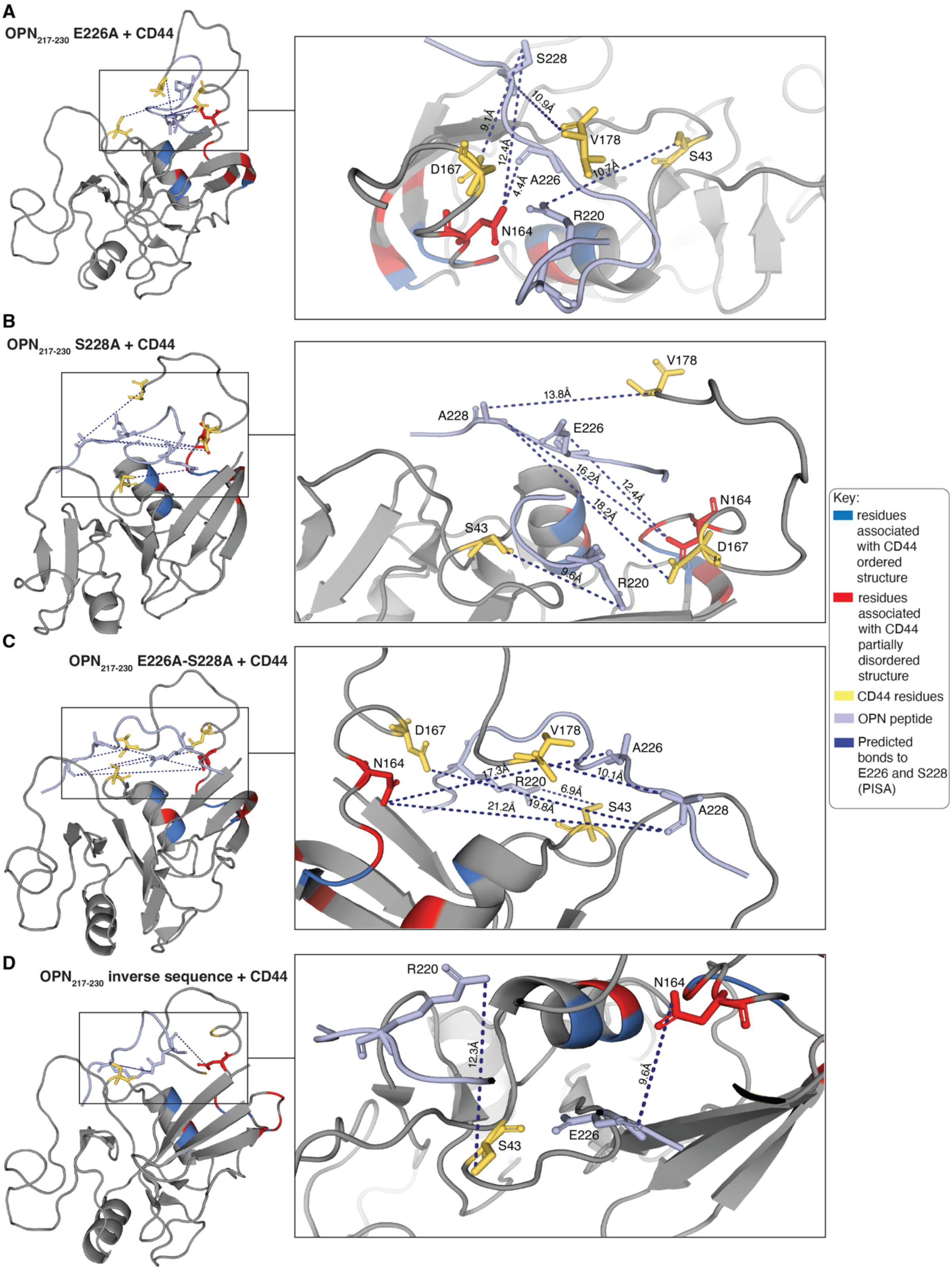

Supplementary figure 6. Prediction of the binding of OPN$_{217-230}$ mutated peptides to CD44. (A-D) Prediction of the docking of OPN$_{217-230}$ E226A (**A**), S228A (**B**), E226A/S228A (**C**) and inverse sequence (**D**) to CD44 ordered conformation (PDB: 1POZ) is shown. Mutated OPN peptides are farther from CD44 S_{43}, N_{164}, D_{167} and V_{178} residues compared to wild type counterpart.
